# Cardiac involvement in Caucasian patients with pulmonary sarcoidosis

**DOI:** 10.1186/1465-9921-15-15

**Published:** 2014-02-07

**Authors:** Pernilla Darlington, Anders Gabrielsen, Peder Sörensson, Kerstin Cederlund, Anders Eklund, Johan Grunewald

**Affiliations:** 1 Respiratory Medicine Unit, Department of Medicine Solna, Karolinska Institutet and Karolinska University Hospital, Stockholm, Sweden; 2 Respiratory Medicine Unit, Department of Medicine, Karolinska Institutet and Södersjukhuset, Sjukhusbacken 10, 118 83 Stockholm, Sweden; 3 Cardiology Unit, Department of Medicine Solna, Karolinska Institutet and Karolinska University Hospital, Stockholm, Sweden; 4 Department of Clinical Science, Intervention and Technology (CLINTEC) at Karolinska Institutet, Stockholm, Sweden; 5 Department of Radiology, Karolinska University Hospital in Huddinge, Stockholm, Sweden

**Keywords:** Cardiac sarcoidosis, Extra-pulmonary involvement, Cardiovascular magnetic resonance, Human leukocyte antigen, Sarcoidosis

## Abstract

**Background:**

Cardiac sarcoidosis (CS) is a potentially life-threatening condition. At present, there is no consensus with regard to the optimal non-invasive clinical evaluation and diagnostic procedures of cardiac involvement in patients with sarcoidosis. The aim of this study in a large homogenous Scandinavian sarcoidosis cohort was therefore to identify risk factors of cardiac involvement in patients with sarcoidosis, and the value of initial routine investigation with ECG and cardiac related symptoms in screening for CS.

**Methods:**

In this retrospective study a cohort of 1017 Caucasian patients with sarcoidosis were included. They were all screened with ECG at disease onset and investigated for CS according to clinical routine.

**Results:**

An abnormal ECG was recorded in 166 (16.3%) of the 1017 patients and CS was later diagnosed in 22 (13.2%) of them, compared to in one (0.1%) of the 851 sarcoidosis patients with a normal ECG (p < 0.0001). The risk for CS was higher in patients with a pathologic ECG combined with cardiac related symptoms (11/40) (27.5%) compared to those with pathologic ECG changes without symptoms (11/126) (8.7%) (p < 0.01). Furthermore, patients with Löfgren’s syndrome had a reduced risk for CS compared to those without (p < 0.05) the syndrome.

**Conclusions:**

This study on an unusually large and homogenous sarcoidosis population demonstrate the importance of an abnormal ECG and cardiac related symptoms at disease onset as powerful predictors of a later diagnosis of cardiac sarcoidosis. In contrast, CS is very rare in subjects without symptoms and with a normal ECG. This knowledge is of importance, and may be used in a clinical algorithm, in identifying patients that should be followed and investigated extensively for the presence of CS.

## Introduction

Sarcoidosis is an inflammatory disease affecting the lungs and/or intrathoracic lymph nodes in 90% of the cases. It is characterized by the formation of non-necrotizing granulomas in the affected organs and almost any organ can be affected leading to a highly variable clinical presentation [1,2]. In patients with cardiac sarcoidosis (CS) is the interventricular septum commonly engaged, leading to damage of the conduction system [3]. Engagement of the heart may therefore manifest itself as benign arrhythmias or give rise to severe conduction blocks and in worst case life threatening arrhythmias secondary to infiltration of the cardiac conduction system or the myocardium [4]. Glucocorticoid treatment is usually recommended in patients with CS to suppress the inflammation and disease progression [5]. CS is believed to occur in about 5% of all patients with sarcoidosis, but heart biopsy samples from deceased patients with a diagnosis of sarcoidosis indicate that the incidence of cardiac engagement is likely to be higher [6,7]. Studies of autopsy materials also show that cardiac involvement is considerably more common in Japanese patients than in Caucasians, indicating that ethnic/genetic factors might be of significant importance [8].

Although a positive cardiac biopsy, with sarcoid granulomas is considered diagnostic for CS, it is highly invasive and difficult to obtain. In addition, a negative biopsy does not exclude CS. At present, there is no consensus with regard to the optimal non-invasive clinical evaluation and diagnostic procedures of patients with suspected cardiac involvement in sarcoidosis, and thus improvement in patient evaluation strategies might help in defining the patients at high risk of cardiac involvement [9].

The overall objective of this study of a large cohort of well-characterized Scandinavian patients with a primary diagnosis of sarcoidosis was to investigate the value of resting electrocardiogram (ECG) and clinical symptoms in the early identification of CS. In addition, different phenotypic and genotypic factors were included in the analysis.

## Materials and methods

### Study subjects

In this retrospective study, 1017 consecutively recruited sarcoidosis patients of Caucasian origin were included (Table 1). The majority were recruited from a single center in Stockholm, Sweden, and were screened with ECG at disease onset. All patients had initially been referred for a first diagnostic investigation and activity assessment of sarcoidosis. Patients with non-resolving disease, i.e. signs of activity according to the criteria defined by Costabel et al. [10], were followed for several years and investigated for CS upon clinical suspicion of heart engagement at later timepoints if deemed indicated. All patients further investigated for CS were examined in line with the routine at the out-patient clinic (see below). All patients had been diagnosed with sarcoidosis through typical clinical and chest radiographic manifestations, findings at bronchoscopy with bronchoalveolar lavage (BAL) including an elevated CD4/CD8 cell ratio (>3.5), and positive biopsies, using the criteria outlined by the World Association of Sarcoidosis and other Granulomatous disorders (WASOG) [7]. Patients were defined as ever smokers if they had previously smoked or were current smokers. Chest radiographs were evaluated and findings staged by one of the authors (chest radiologist K. C.). Chest radiographs in patients with sarcoidosis were classified into five stages: Stage 0 - normal; Stage I - bilateral hilar lymphadenopathy; Stage II - bilateral lymphadenopathy with parenchymal infiltrates; Stage III – parenchymal infiltrates alone; Stage IV - fibrotic bands and volume reduction [11]. Patients described as having parenchymal lung infiltrates included them with stage II-IV. Löfgren’s syndrome (LS) (n = 386) was defined as bilateral hilar lymphadenopathy with or without parenchymal infiltration, fever, erythema nodosum and/or ankle arthritis. An evaluation of deceased patients was performed in August 2013. This was possible since patient records do simultaneously operate with the tax authority system registrating all citizens. Data was missing for those who were no longer residents in Sweden.

**Table 1:** Clinical characteristics of 1017 patients

	**Patients with pathologic ECG**	**Patients with normal ECG**
Subjects	166	851
Gender M/F	107/59*	473/378
Age, years^^^	41 (25–76)	38 (9–78)
Smoker		
(never/ever/unknown)	99/67/0	467/370/14
Radiographic stage		
0/I/II/III/IV	8/63/57/30/8^##^	57/426/273/74/21
Löfgren/non-Löfgren	48/118**	338/513
Alive/deceased/unknown	150/14/2^#^	817/31/3

*p < 0.05 compared to the ratio of male/female gender among patients with a normal ECG. ^^^Age, years at time for diagnosis: values are medians (min – max). ^##^p < 0.01 compared to the ratio of stage II-IV among patients with a normal ECG. **p < 0.01 compared to the ratio of LS/non-LS patients with a normal ECG. ). ^#^p < 0.05 compared to the ratio of alive/diseased with a normal ECG.

Patient characteristics for the whole group are described in Table 1, divided into patients with a pathologic ECG and those with a normal ECG. The frequency of ECG changes judged as pathological are described in Table 2. The diagnosis CS was guided by the Japanese guidelines by Hiraga et al. [12] in the vast majority of cases. A few subjects did not fulfill all the criteria in the guidelines but they were regarded having CS after careful cardiological investigation. An additional magnetic resonance imaging (MRI) had not been performed in all patients since this has become a routine only recently. All patients diagnosed with CS were evaluated by one of the authors (cardiologist A. G.). Characteristics for patients finally diagnosed with CS are described in Table 3. Written informed consent was obtained from all subjects, and the study was approved by the Regional Ethical Review Board in Stockholm, Sweden. All except three patients had their human leukocyte antigen (HLA)-DRB1 analyzed.

**Table 2:** Frequency (%) of different symptoms and changes regarded as pathologic on ECG in patients with an abnormal ECG (n = 166)

Symptoms	Palpitations	18.1
	Pre-syncope	2.4
	Syncope	3.6
Pathologic changes	Atrioventricular block	9.6
	Right bundle branch block	13.9
	Incomplete right bundle branch	
	Block	4.2
	Left bundle branch block	2.4
	Left anterior fascicular block	13.9
	Unspecific bundle branch block	1.2
	Premature ventricular contractions	15.1
	Atrial flutter	1.8
	Pathologic Q waves and ST-T changes	44.6

**Table 3:** Clinical characteristics of patients diagnosed with CS

Subjects	23
Gender M/F	15/8
Age, years^	44 (26–62)
Age, years^#^	48 (28–75)
Smoker (never/ever)	13/10
Radiographic stage	4/7/7/5/0
0/I/II/III/IV	
Löfgren/non-Löfgren	3/20*
Alive/deceased/unknown	22/1

^Age, years at time for sarcoidosis diagnosis: values are

medians (min – max). ^#^Age, years at time for diagnosis of CS:

values are medians (min – max). *p < 0.05 compared to the ratio of LS/non-LS

patients without CS.

### HLA typing

HLA-class II (HLA-DR) typing was done on DNA from blood samples with the polymerase chain reaction (PCR) and amplification with sequence-specific primers (SSP) [13].

### Statistical analysis

Data were analyzed by Chi-square test or in the case of small numbers by Fisher’s Exact Test. Relative risk (RR) was calculated from the cross product ratio of the entries in Table 1 and for Table 3 with patients without CS. Statistical analyses and graphs were performed with Graph Pad Prism 6 (GraphPad Software Inc., San Diego, CA, USA). P-value <0.05 was regarded as significant except from the comparison of different allele frequencies, where p < 0.003 (p < 0.05/13) was regarded as significant [after Bonferroni correction for the number of alleles (n = 13)]. Sensitivity, specificity, positive and negative predictive values of ECG-screening were calculated considering a patient with a pathologic ECG diagnosed with CS as true positive and false positive if the ECG was pathologic but CS was not diagnosed during the follow up.

## Results

Out of the 1017 included patients there were 166 (16.3%) patients with an abnormal ECG. Among these patients there were more males (RR) (1.4) (p < 0.05), more patients with parenchymal lung infiltrates on chest radiography (stage II-IV) (1.6) (p < 0.01), less patients with LS (0.7) (p < 0.01) and more patient deaths (2.3) (p < 0.05) compared to patients with a normal ECG (Table 1).

There was a more detailed cardiac investigation performed in the majority of the 166 patients with a pathologic ECG (69.9%, n = 116) as well as in 21.2% (n = 180) of the 851 patients with a normal ECG, but with cardiac related symptoms such as palpitations, pre-syncope and syncope. Also patients with dyspnea, chest pain and examination findings such as heart murmur were included in this group. Included among the 166 patients with an abnormal ECG, there were three patients with CS that developed a pathologic ECG and symptoms indicative of CS during the follow-up, but who initially had a normal ECG and were asymptomatic in regard to cardiac syptoms. In the group of patients with a pathological ECG, 22 patients were diagnosed with CS during the follow up. One patient diagnosed with CS had a normal ECG but a pathologic 24-hour ambulatory ECG. In addition, 15 patients among those with a pathologic ECG and six with initially a normal ECG were diagnosed with cardiac disease, however judged not to be related to sarcoidosis. These were patients with ischemic heart disease (n = 6), valvular heart disease (n = 4) and aortic aneurysm (n = 1). There were also nine patients with atrial fibrillation and one patient with pacemaker that had not received the diagnosis of CS (Table 4).

**Table 4:** Clinical characteristics of patients diagnosed with other cardiac disease than CS

Subjects	21
Gender M/F	14/7
Age, years^	44 (29–78)
Age, years^#^	54 (9–81)
Smoker (never/ever)	16/5
Radiographic stage	1/13/2/4/1
0/I/II/III/IV	
Löfgren/non-Löfgren	5/16
Alive/deceased/unknown	18/3

^Age, years at time for sarcoidosis diagnosis: values are

medians (min – max). ^#^Age, years at time for diagnosis of cardiac disease:

values are medians (min – max).

Five of the 23 patients diagnosed with CS had a normal TTE. An additional CMR could not be performed in four of these patients as they urgently received a pacemaker because of severe conduction disturbances. The fifth exhibited pathologic ECG changes that disappeared after treatment with steroids. All of them had an established sarcoidosis diagnosis; two of them had classical Löfgrens syndrome and the other three had a positive biopsy that confirmed the diagnosis. They were, however, incompletely investigated in terms of all the criteria set forth in the guidelines but were diagnosed with CS based on the combination of CS criteria present and their clinical presentation. Twenty-two (13.2%) of 166 patients with an abnormal ECG were found to have CS, compared to one (0.1%) of the 851 patients with a normal ECG (p < 0.0001). According to the results including all 1017 patients, the sensitivity of a pathologic ECG to predict CS was 95.6% and the specificity was 85.5%. The positive predictive value of a pathologic ECG was 13.3% and the negative predictive value of a normal ECG was 99.9%.

**Figure 1:**
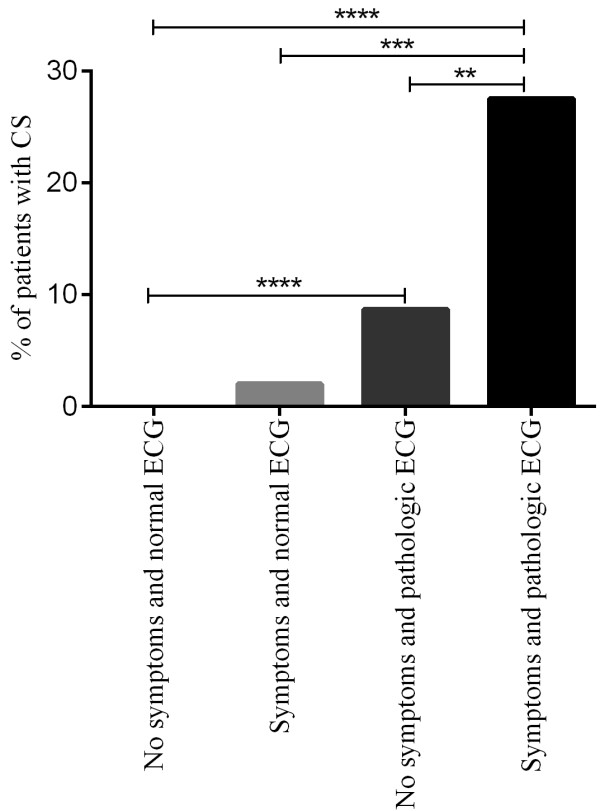
**Frequency of CS in patients divided into four groups: patients with no symptoms and a normal ECG (n = zero out of 800), patients with symptoms but a normal ECG (n = one out of 51), patients with no symptoms but a pathologic ECG (n = 11 out of 126) and patients with symptoms and a pathologic ECG (n = 11 out of 40).** Symptoms are palpitations, pre-syncope and syncope. **p < 0.01, ***p < 0.001 and ****p < 0.0001.

The greatest risk for CS was observed in patients presenting with a pathologic ECG combined with cardiac related symptoms such as palpitations, pre-syncope and syncope where 11 out of 40 (27.5%) had CS. In these patients the risk was significantly higher compared to patients with an abnormal ECG but without cardiac related symptoms (11/126) (8.7%) (p < 0.01) (Figure 1). Only one of the patients with CS who had severe heart failure died during the median time of follow-up of 7 years (min – max, 1–38 years) at an age of 77 in what was regarded as a sarcoidosis related death. A second patient with severe heart failure had a successful cardiac transplantation. Two patients received an implantable cardioverter-defibrillator (ICD) because of ventricular tachycardias and eight patients needed a pacemaker. The only patient with a normal ECG and CS had symptoms with palpitations and a pathologic 24-hour ambulatory ECG.

The average age in patients ultimately diagnosed with CS was 44 years, four years after the diagnosis of sarcoidosis was first established. There was a tendency within the group of CS of more males than females (RR) (1.4), and also a higher frequency (1.3) of patients with parenchymal lung infiltrates (stage II-IV) (statistically non-significant) (Table 3). LS was significantly less common in patients with CS compared to patients without the syndrome (0.2) (p < 0.05). Smoking as evaluated by comparing never and ever-smokers did not seem to significantly influence the risk for CS.

## Discussion

This study on an unusually large and homogenous sarcoidosis population demonstrate the importance of an abnormal ECG and cardiac related symptoms at disease onset as powerful predictors of a later diagnosis of cardiac sarcoidosis. This knowledge is of importance and may be used in a clinical algorithm, in identifying patients that should be followed and investigated extensively for the presence of CS.

As described, we found a significantly increased risk for CS in sarcoidosis patients presenting with a pathologic ECG in particular if they simultaneously had symptoms that could be cardiac related such as palpitations, pre-syncope or syncope. Conversely, a patient with a normal ECG and without any of the above mentioned cardiac related symptoms had a very low risk of developing CS. Interestingly, we did however not observe any sudden cardiac deaths during the follow-up of these patients and only one death due to severe heart failure. It has been debated whether the diagnosis of CS per se is an indication for ICD implantation to prevent arrhythmic sudden cardiac death [14]. Our observations from this study in Scandinavian Caucasians patients, suggest that diagnosis of CS does not necessarily constitute and independent indication for an ICD implantation, but should probably be evaluated on a patient-by-patients basis.

Based on our experiences in patients with sarcoidosis we also noted that, absence of or “non-significant” ECG changes at the initial screening may later progress and repeated examinations with ECG may therefore be required [5]. As our results show, there were phenotypic factors associated with a pathological ECG as well as with CS. There was a tendency for a male dominance among patients diagnosed with CS, which differs slightly from what was reported by Iwai et al. in a previous study of autopsy material from deceased sarcoidosis patients [8]. In Caucasians, they observed an almost equal number of males and females with CS, but in a Japanese cohort, however, they reported that cardiac involvement was more common in females. Our investigation does not offer any explanation(s) as to why we observe more males than females diagnosed with CS, but it must be remembered that our cohort is diagnosed clinically and alive as opposed to autopsy diagnosis, and direct comparisons between live and deceased patient cohort should be done with caution. In our cohort there was a predominance of males among all patients with sarcoidosis leading to an inherent gender bias to the cohort. Therefore, from the results of our investigation, it is not possible to firmly establish or refute that gender might influence the risk of CS differently in different ethnic groups or that gender may have different impact on morbidity and mortality. Furthermore, never-smokers out-numbered smokers in both patients with and without CS. An increased risk for a pathological ECG, and a similar tendency of an increased risk for CS, was seen in patients with parenchymal lung infiltrates on chest radiography. Patients with LS had a significantly reduced risk for CS compared to non-LS patients. In line with that LS patients have a very favorable prognosis [15].

This is a retrospective cohort study, and although it is a large cohort, the retrospective nature and other inherent sources of bias may present a limitation to the interpretation. First, the frequency of CS in the whole study population may have been higher than the one we observed if more or all patients with or without a pathological ECG had been investigated in a more extensive way than was possible in this historic cohort. However, we do not believe that the essential observation of a normal ECG at baseline as a high negative predictor of CS would be challenged. Secondly, our observations are based on the clinical decision making and diagnosis and not on adjudication committee diagnosis, contributing to to less standardization. In this investigation patients were followed from their onset of illness (few months prior to first visit at our clinic) with an active disease and followed as long as they exhibited signs of disease activity. Thus all patients diagnosed with CS had an active disease. Because of the clinical decision making variations in cardiac investigations over time, we had to include patients diagnosed with CS (clinical decision of a very high risk of CS) exhibiting a normal TTE and who had not been investigated with MRI. We believe that these patients should be included in the CS group based on the clinical phenotype and a previous report by Metha et al. demonstrating a pathologic TTE in only 25% of the patients finally diagnosed with CS [16]. Similarly, one of the patients in our material diagnosed with CS had a normal resting ECG but a pathologic twenty-four-hour ambulatory ECG. Suzuki et al. reported that a high frequency of VES was more common in patients with CS than in those without [17], and we believe that this patient should be clinically diagnosed with CS. However, we do emphasize that these above mentioned patients are only a minor part of the total number of patients included. Therefore, it is expected that minor errors may be introduced in the incidence-estimates but the basic conclusions from this observational study is not expected to be over-ruled.

In conclusion, our results show that the risk for CS is significantly higher in sarcoidosis patients presenting with a pathologic ECG and in particular if they simultaneously exhibit cardiac related symptoms such as palpitations, pre-syncope and syncope. In accordance with our findings we recommend that all sarcoidosis patients who have a pathologic ECG should be further investigated for CS. Patients who have a normal ECG at first presentation and lack the above symptoms of suspected cardiac involvement appear to be at a low risk for CS. Follow-up with repeated ECGs at regular intervals appears to be motivated particularly in patients with non-LS disease and should enhance the possibility to detect CS at an early stage, and thereby to intervene and prevent severe cardiac related complications.

## Competing interests

The authors declare that they have no competing interest.

## Authors’ contributions

PD designed and coordinated the study, wrote the application to the Ethics Committee, characterized patients, summarized data and drafted the manuscript. AG co-designed the study and characterized patients, interpreted data and helped writing the manuscript. PS characterized patients and helped writing the manuscript. KC classified radiographs. AE co-designed the study and characterized patients, interpreted data and helped writing the manuscript. JG co-designed the study and characterized patients, interpreted data and helped writing the manuscript. All authors read and approved the final manuscript.

## References

[B1] Newman LS Rose CS Maier LA Medical progress - sarcoidosis N Engl J Med 1997 336 1224 1234 10.1056/NEJM199704243361706 9110911

[B2] Iannuzzi MC Rybicki BA Teirstein AS Medical progress: sarcoidosis N Engl J Med 2007 357 2153 2165 10.1056/NEJMra071714 18032765

[B3] Matsui Y Iwai K Tachibana T Fruie T Shigematsu N Izumi T Homma AH Mikami R Hongo O Hiraga Y Yamamoto M Clinicopathological study of fatal myocardial sarcoidosis Ann N Y Acad Sci 1976 278 455 469 10.1111/j.1749-6632.1976.tb47058.x 1067031

[B4] Fleming HA Cardiac sarcoidosis Clin Dermatol 1986 4 143 149 10.1016/0738-081X(86)90044-1 3542169

[B5] Nunes H Freynet O Naggara N Soussan M Weinman P Diebold B Brillet PY Valeyre D Cardiac sarcoidosis Semin Respir Crit Care Med 2010 31 428 441 10.1055/s-0030-1262211 20665393

[B6] Silverman KJ Hutchins GM Bulkley BH Cadiac sarcoid - clinicopathologic study of 84 unselected patients with systemic sarcoidosis Circulation 1978 58 1204 1211 10.1161/01.CIR.58.6.1204 709777

[B7] Hunninghake GW Costabel U Ando M Baughman R Cordier JF du Bois R Eklund A Kitaichi M Lynch J Rizzato G Statement on sarcoidosis Am J Respir Crit Care Med 1999 160 736 75510430755

[B8] Iwai K Sekiguti M Hosoda Y Deremee RA Tazelaar HD Sharma OP Maheshwari A Noguchi TI Racial difference in cardiac sarcoidosis incidence observed at autopsy Sarcoidosis 1994 11 26 31 8036339

[B9] Mantini N Williams B Jr Stewart J Rubinsztain L Kacharava A Cardiac sarcoid: a clinician’s review on how to approach the patient with cardiac sarcoid Clin Cardiol 2012 35 410 415 10.1002/clc.21982 22499155 PMC6652737

[B10] Consensus conference: activity of sarcoidosis. Third WASOG meeting, Los Angeles, USA, September 8–11, 1993 Eur Respir J 1994 7 624 627 8013622

[B11] Scadding JG Prognosis of intrathoracic sarcoidosis in England - a review of 136 cases after 5 years observation Br Med J 1961 2 1165 1172 10.1136/bmj.2.5261.1165 14497750 PMC1970202

[B12] Hiraga H Hiroe M Iwai K Guideline for diagnosis of cardiac sarcoidosis: study report on diffuse pulmonary diseases (in Japanese) The Japanese Ministry of Health and Welfare 1993 23 24

[B13] Olerup O Aldener A Fogdell A HLA-DQB1 and HLA-DQA1 typing by PCR amplification with sequence-specific primers (PCR-SSP) in 2 hours Tissue Antigens 1993 41 119 134 10.1111/j.1399-0039.1993.tb01991.x 8316943

[B14] Kim JS Judson MA Donnino R Gold M Cooper LT Prystowsky EN Prystowsky S Cardiac sarcoidosis Am Heart J 2009 157 9 21 10.1016/j.ahj.2008.09.009 19081391

[B15] Grunewald J Eklund A Lofgren’s syndrome human leukocyte antigen strongly influences the disease course Am J Resp Crit Care Med 2009 179 307 312 10.1164/rccm.200807-1082OC 18996998

[B16] Mehta D Lubitz SA Frankel Z Wisnivesky JP Einstein AJ Goldman M Machac J Teirstein A Cardiac involvement in patients with sarcoidosis: diagnostic and prognostic value of outpatient testing Chest 2008 133 1426 1435 10.1378/chest.07-2784 18339784

[B17] Suzuki T Kanda T Kubota S Imai S Murata K Holter monitoring as a noninvasive indicator of cardiac involvement in sarcoidosis Chest 1994 106 1021 1024 10.1378/chest.106.4.1021 7523035

